# Simultaneous Detection of Ammonium and Nitrate in Environmental Samples Using on Ion-Selective Electrode and Comparison with Portable Colorimetric Assays

**DOI:** 10.3390/s18103555

**Published:** 2018-10-19

**Authors:** Jittima Choosang, Apon Numnuam, Panote Thavarungkul, Proespichaya Kanatharana, Tanja Radu, Sami Ullah, Aleksandar Radu

**Affiliations:** 1 The Birchall Centre, Lennard-Jones Laboratories, Keele University, Keele ST5 5BG, UK; Jittima_ch@hotmail.com; 2 Trace Analysis and Biosensor Research Center, Prince of Songkla University, Hat Yai 90112, Thailand; anumnuam@gmail.com (A.N.); panote.t@psu.ac.th (P.T.); proespichk@gmail.com (P.K.); 3 Department of Chemistry, Faculty of Science, Prince of Songkla University, Hat Yai 90112, Thailand; 4 Department of Physics, Faculty of Science, Prince of Songkla University, Hat Yai 90112, Thailand; 5 School of Architecture, Building and Civil Engineering, Loughborough University, Ashby Road, LE113TU Loughborough, Leicestershire, UK; t.radu@lboro.ac.uk; 6 School of Geography, Earth and Environmental Sciences and Birmingham Institute of Forest Research, University of Birmingham, B15 2TT Birmingham, Edgbaston, UK; S.Ullah@bham.ac.uk

**Keywords:** ion-selective electrodes, environmental analysis, soil analysis, electrodes versus colorimetric assay

## Abstract

Simple, robust, and low-cost nitrate- and ammonium-selective electrodes were made using substrate prepared from household materials. We explored phosphonium-based ILs and poly (methyl methacrylate)/poly(decyl methacrylate)(MMA-DMA) copolymer as matrix materials alternative to classical PVC-based membranes. IL-based membranes showed suitability only for nitrate-selective electrode exhibiting linear concentration range between 5.0 × 10^−6^ and 2.5 × 10^−3^ M with a detection limit of 5.5 × 10^−7^ M. On the other hand, MMA-DMA—based membranes showed suitability for both ammonium- and nitrate-selective electrodes, and were successfully applied to detect NO_3_^−^ and NH_4_^+^ in water and soil samples. The proposed ISEs exhibited near-Nernstian potentiometric responses to NO_3_^−^ and NH_4_^+^ with the linear range concentration between 5.0 × 10^−5^ and 5.0 × 10^−2^ M (LOD = 11.3 µM) and 5.0 × 10^−6^ and 1.0 × 10^−3^ M (LOD = 1.2 µM), respectively. The power of ISEs to detect NO_3_^−^ and NH_4_^+^ in water and soils was tested by comparison with traditional, portable colorimetric techniques. Procedures required for analysis by each technique from the perspective of a non-trained person (e.g., farmer) and the convenience of the use on the field are compared and contrasted.

## 1. Introduction

Fertilizer use has been essential to feed half of the world’s population over the 20th century and will be fundamental to ensure global food security over the 21st century. Interestingly, approximately 2% of world energy use is dedicated to the industrial manufacture of reactive nitrogen (N_r_) mainly through the Haber-Bosch process to produce ammonia. Worryingly, the efficiency of nutrient use is very low: on average over 75% of added nutrients end up lost to the environment, thus causing pollution either of water systems or through emissions of highly potent greenhouse gases N_2_O and NH_3_, and carcinogens such as NO_2_. One report suggests that a 20% improvement in nutrient use efficiency would reduce use of nitrogen fertilizer by 20 million tonnes annually. This in turn could produce a net saving worth around £110 billion per annum [1].

Nitrogen is used by plants in the forms of nitrate (NO_3_^−^) and ammonium (NH_4_^+^) [2]. These ions are produced by nitrification process; NO_3_^−^ production includes ammonium (NH_3_) oxidation to nitrite (NO_2_^−^) followed by NO_2_^−^ oxidation to NO_3_^−^. On the other hand, NH_4_^+^ in soil is produced by the reduction of NO_3_^−^ [3]. So, these ions can exist together in soil due to the use of fertilizers in soil, agricultural cultivation, nitrogen fixation, and animal waste [4]. While being extremely useful in agriculture due to their leaching, NO_3_^−^ and NH_4_^+^ ions in soil may cause pollution and become a major ecological problem in agricultural areas. For example, concentrations of NO_3_^−^ in water are taken as indicators of water quality. The Ministry of Health with the European Council sets the highest limiting value of nitrate 50 mg·L^−1^ and 15 mg·L^−1^ in drinking water and water for infants, respectively [5]. Therefore, simultaneous determination of NO_3_^−^ and NH_4_^+^ in soil samples is very important in order to control and optimize nutrient availability for plants and determine production and consumption rate related to the N cycle. Various conventional techniques are used to routinely detect NO_3_^−^ and NH_4_^+^ such as liquid chromatography (IC), flow injection analysis (FIA), colorimetric assay etc. A recent report demonstrates the use of electrochemical impedance spectroscopy (EIS)-based sensors for direct sensing of NO_3_^−^ in a controlled tree nursery environment and in a certified growing medium [6]. However, these techniques have some drawbacks. For example, farmers have very limited access to IC and FIA due to their cost and complexity of operation [7]. Even colorimetric assays that are typically marketed as suitable for in field use are typically used for research purposes. In our admittedly limited and anecdotal experience, farmers perceive colorimetric assays as complex and expensive. We will analyze procedures for sample analysis by both techniques later in the text. Therefore, the sensitive, simple and inexpensive method is highly required for NO_3_^−^ and NH_4_^+^ detection.

Ion selective electrodes (ISEs) are an alternative chemical sensor to detect the analytes which are easy to manufacture, show excellent sensitivity and selectivity, and ability to connect to simple communication devices [8,9,10]. ISEs are have been widely used in many fields (most significantly in clinical analysis, although applications in agriculture, food processing, and pollution monitoring are relevant too). Practitioners intending to apply ISEs in the field are typically critical of ISEs’ robustness and precision [11]. A significant amount of work has been done in recent years to address some of the common criticism [12,13,14,15,16]. Reducing the complexness of operation with ISEs with the view of reducing farmers’ distrust of analytical instrumentation is in a focus of our group. For example, we have very recently demonstrated a very simple substrate for ISEs that uses only materials available in common households [17].

We strongly believe that materials will continue to have major role in developing extremely simple sensing devices while in the same time significantly improving their functionality. For example, conductive polymers and carbon nanotubes has pushed boundaries of ISEs development [18,19]. In another example, acrylate-based polymers have enabled excellent improvements in ISEs while ionic liquids (ILs) are coming into fashion [20,21]. The later show promise due to almost unlimited structural differences thus changing functionality and analytical performance of ISEs [22]. Interestingly, currently there is a debate on actual role of ILs, as they have been successfully utilized as almost any major component of ISEs (ionophore, ion exchanger, plasticizer) [23,24,25]. Acrylate-based ISEs showed significant improvements sensitivity and selectivity over some of the traditionally used materials [26,27]. For example, a plasticizer-free polymer membrane composition based on a methyl methacrylate and decyl methacrylate (MMA-DMA) copolymer proposed by Bakker’s group [28,29] exhibited improvement in sensing characteristics, while also improving the simplicity of sensor preparation and handling [30] has thus emerged as an excellent material for demonstrating ultra-simple sensors for in field use.

Herein, we study the role and the influence of two types of phosphonium-based ILs: (trihexyl(tetradecyl)phosphoniumbis(trifluoromethanesulfonyl) [P_6,6,6,14_][TFMS] and trihexyl(tetradecyl) phosphoniumdicyanamide[P_6,6,6,14_][DCA]). We use them as plasticizers in view of improvement of robustness and sensing properties of ISEs. In addition, we utilized MMA-DMA to prepare ISEs that require almost no handling prior- and post-operation with the view of development of sensors that farmers can deploy on their fields. For this purpose, we utilized our substrate made of house-hold materials. We hope to develop sensors that will be sufficiently sensitive and robust, and that can be used at home by non-trained personnel (e.g., farmers). Ideally, they will be very simple for handling, thus reducing farmers’ stress and wariness of complex analytical instrumentation. We demonstrated our sensors in direct, simultaneous analysis of NO_3_^−^ and NH_4_^+^ in a number of agriculturally-relevant soil and water samples.

## 2. Experimental

### 2.1. Reagents

Ionic liquids including trihexyl (tetradecyl) phosphoniumbis (trifluoromethanesulfonyl) [P_6,6,6,14_] [TFMS] and trihexyl (tetradecyl) phosphoniumdicyanamide [P_6,6,6,14_][DCA] were purchased from Strem chemicals (purities > 95%, Cambridge, UK), Tetradodecylammonium chloride (TDACl), poly(vinyl chloride) (PVC), bis(2-ethylhexyl)sebacate (DOS)(Fluka), Nonactin (ammonium ionophore I), and tetradodecylammonium tetrakis(4-chlorophenyl)borate (ETH 500) were obtained from Sigma-Aldrich (Gillingham, UK). Methyl methacrylatedecyl methacrylate (MMA-DMA) copolymer matrix and sodium tetrakis[3,5-bis- (trifluoromethyl)-phenyl]borate (NaTFPB) were obtained from Euvive (Aurora, CO 80045, USA). Tetrahydrofuran (THF) was obtained from Fisher (Loughborough, UK). All aqueous solutions were prepared in ultrapure water obtained from Pico Pure 3 water system.

### 2.2. Preparation of Pencil-Drawn Electrodes

**Scheme 1:**
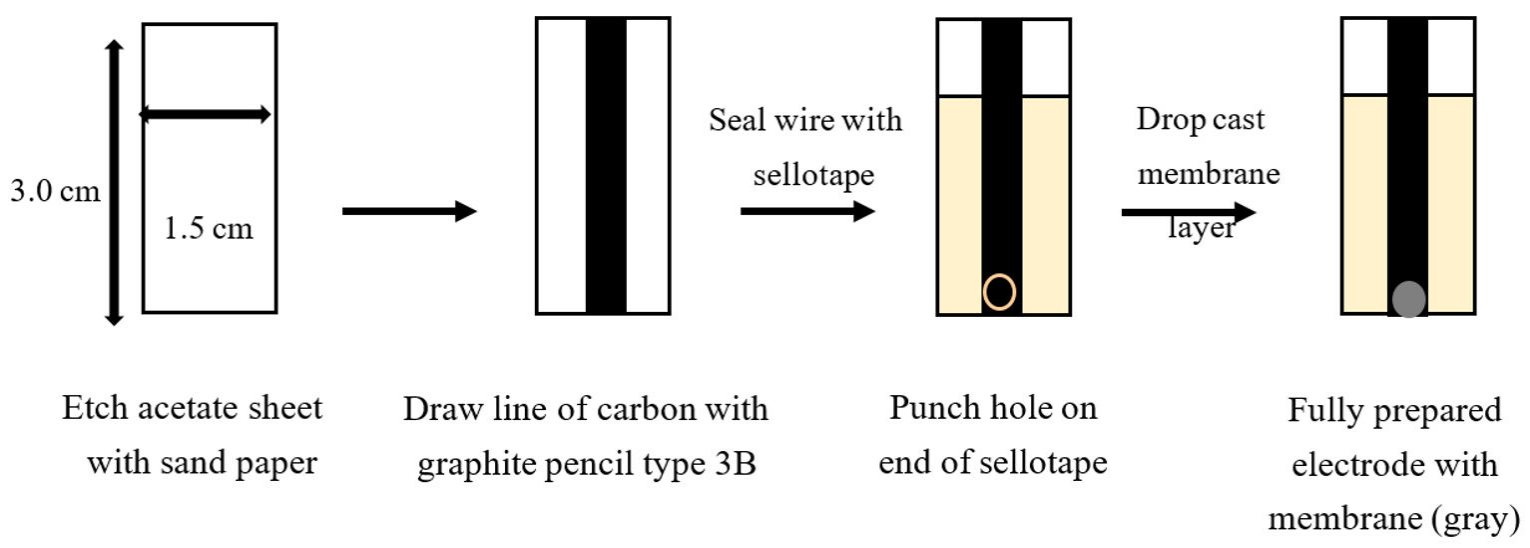
Schematic representation of pencil-drawn ion selective electrode.

The pencil-drawn electrodes were prepared as described previously [17]. Briefly, a cellulose acetate sheet, otherwise known under commercial name ‘acetate’ (used in variety of hobbies and available in most bookstores), was cut in small pieces (1.5 × 3.0 cm) to form a single electrode. It was then etched with aluminum oxide (grit 240) for 30 s to provide rough surface in order to enable better adhesion of graphite onto its surface. A line is then drawn using regular graphite pencil (type 3B) by hand. The consistency of the line was checked by measuring of its resistance with a simple multimeter. If the resistance was higher than 5 kΩ (or measurements indicated that the line is not continuous), more graphite was added by further abrasion. The line was insulated using sellotape, making sure that the top end is left opened for connection with the instrument via alligator clips. A hole of ~0.30 cm in diameter was punched at the distal end of the sellotape prior adhesion. The sellotape was carefully placed so that the hole aligned with the graphite line. This hole was used to deposit the cocktail which was prepared as described in Section 2.3. The procedure for the preparation of single electrodes is illustrated in Scheme 1, although we have also prepared sensing strips for the simultaneous analysis of NO_3_^−^ and NH_4_^+^ ions, as illustrated earlier [17].

### 2.3. Preparation of Sensing Membrane for Nitrate and Ammonium Detection

Table 1 presents the composition of all membranes used here. Total mass of all membranes was 200 mg. All chemicals of every membranes were immediately dissolved in 1.0 mL of THF, and the resulting cocktail was vortexed for 30 min to complete the dissolution of component. Then, the components solution was drop cast onto the substrate until the membrane thickness is ~200 μm and left it to dry at room temperature overnight. Electrodes were conditioned in 1.0 × 10^−3^ M of NH_4_NO_3_ for 12 h prior the use. Electrodes used for optimizing limit of detection (LOD) underwent additional conditioning step consisting of overnight conditioning in the mixture solution of 1.0 × 10^−8^ of NH_4_NO_3_ and 1.0 × 10^−3^ of NaNO_3_.

### 2.4. EMF Measurements

Potentiometric responses of all electrodes were record using the Lawson Labs Inc (Malvern, PA 19355, USA). 16-channel EMF-16 interface (3217 Phoenixville Pike, Malvern, PA, USA) in the stirred solution against a double-junction Ag/AgCl reference electrode with a 1.0 M of LiOAc bridge electrolyte (Fluka). All ISEs were immersed into 20.0 mL of ultra-pure water in a beaker followed by stepwise addition of known concentration of NH_4_NO_3_. Electrodes were rinsed with ultra-pure water before immersing into the next sample to avoid carryovers. Activity coefficients (log a) were calculated according to the Debye-Hückel approach and voltages were corrected for liquid-junction potentials with the Henderson equation. All measurements are done using at least 6 electrodes of the same kind at a time.

### 2.5. Analysis of NO_3_^−^ and NH_4_^+^ in Soil and Water Samples Using ISEs

#### 2.5.1. Study Sites and Sampling

**Figure 1:**
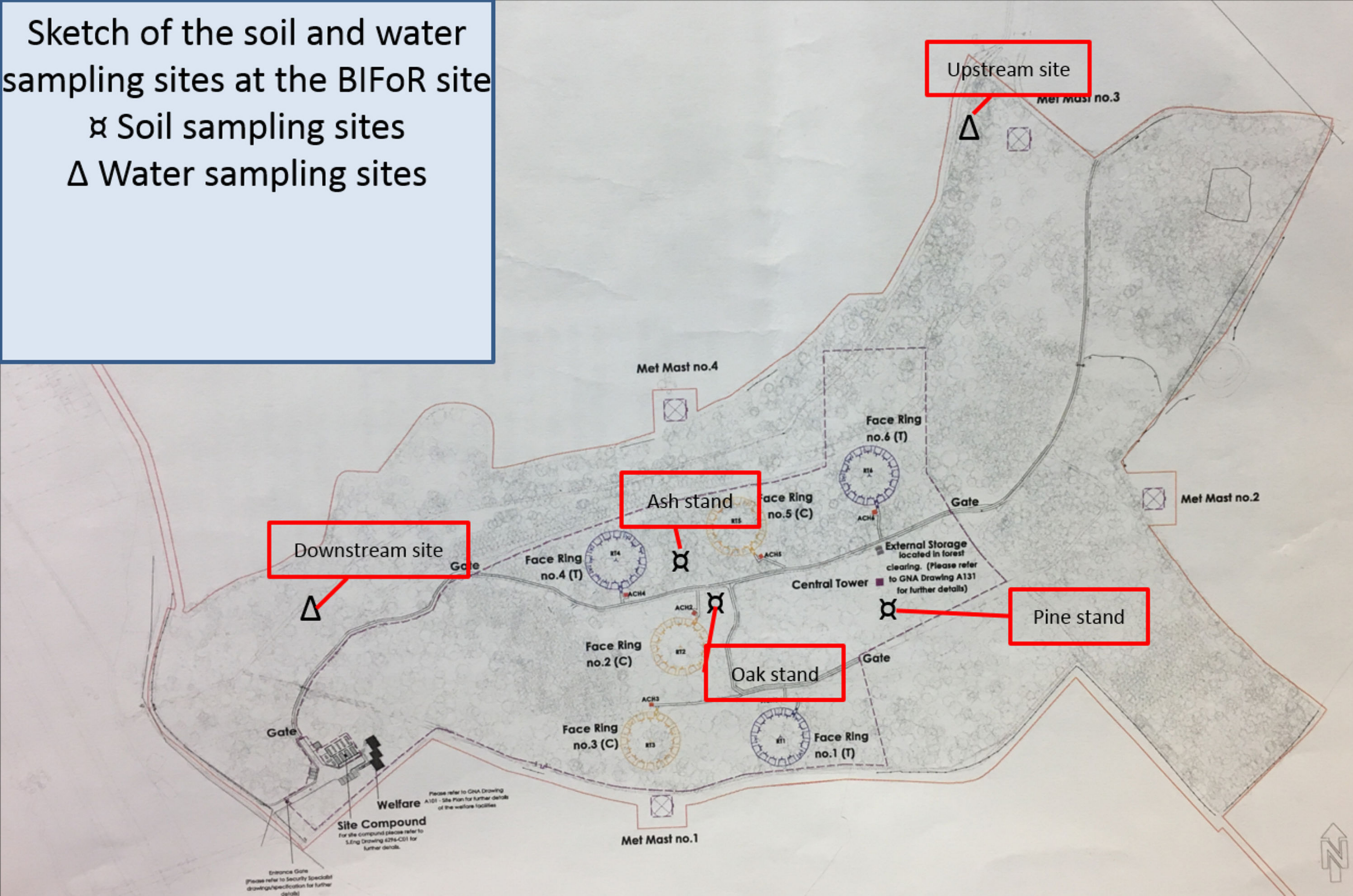
Map of BIFoR site with note water and soil sampling sites. Note that FACE Rings stand for Free-Air Carbon Dioxide Enrichment (FACE). They are a series of approximately cylindrical ring structures, as high as the tree canopy (around 25 m) and 30 m wide, supporting pipes that deliver CO_2_ in such a way that the woodland inside the ring is immersed in elevated CO_2_, but the rest of the woodland remains largely unaffected. For more details, the reader is kindly asked to visit BIFoR’s web site [31].

All environmental water and soil sample were collected at Birmingham Institute for Forest Research (BIFoR) as noted on the map (Figure 1).

We collected a total of 8 water samples (four upstream (US) and four downstream (DS)) from the stream going through the site. We also collected total of 15 soil samples. Five samples were taken from each site rich with Scottish Pine (SP), Ash (ASH), and Oak (OAK).

Prior to sampling of soils, the overlying vegetation cover was removed and one soil core (2–15 cm depth; 5 cm diameter) was collected for each sample plot using a hand auger. The soils were collected, homogenized by manual mixing, and stored in gas permeable polyethylene bags before laboratory analysis. All samples, one travel blank, and two filter blanks were transferred on ice to the laboratory within 2 h of collection, where they were refrigerated at <5 °C until needed for the experimental procedures. Immediately prior to use, the soil was sieved to 4 mm to remove plant materials, large stones, and earthworms and then thoroughly mixed.

Aqueous samples were sampled according to standard water sampling procedure [32,33].

#### 2.5.2. Background Soil Analysis

The main physico-chemical soil analysis were performed on air-dried soils, and according to established methods [34]. Soil moisture content was measured gravimetrically as moisture lost from a subsample of air-dried soils by continuous heating (105 °C) for up to 24 h until constant weight was achieved. Soil pH was measured at (soil:water mix = 1:2.5) by a standard pH probe. For all analysis, samples were blank corrected, and the level of precision was calculated as presented in Table 2.

#### 2.5.3. Extraction Procedure of NH_4_^+^ and NO_3_^−^ from Soil

A standard methodology for extraction of reactive nitrogen (Nr) requires solution of 2 M KCl. Due to the selectivity (please see below), we used 0.1 M MgSO_4_ as a single extracting medium for simultaneous extraction of NH_4_^+^ and NO_3_^−^. In all cases, 20 g of air-dried sieved (<2 mm) soil were weighed into 250-mL HDPE Nalgene bottles. This was followed by extraction of Nr from soil samples using 100 mL of chosen solution. Briefly, the soil slurries (a combination of soil sample and extractant) were continuously shaken on a reciprocating shaker at 200 rpm for 1 h before being centrifuged at 4000 rpm for 30 min, followed by a two-step filtration into 20 mL scintillation vials through a no. 42 Whatman filter paper, and then 0.45 micron syringe filters (Whatman, Cambridge, UK). All analyses were carried out immediately unless otherwise stated, i.e., when samples were frozen until analysis.

#### 2.5.4. Analysis of NH_4_^+^ and NO_3_^−^ Using ISEs

The unknown concentrations of NO_3_^−^ and NH_4_^+^ were determined using the standard addition method. Twenty milliliters of sample solution was spiked with 60.0 µL of stock solution (0.1 M NH_4_NO_3_) until the potential change around 30–50 mV to give final concentration of NO_3_^−^ and NH_4_^+^ by neglecting the original concentration of analyte in samples. The unknown concentration was estimated by using the linear regression analysis. All results of NO_3_^−^ and NH_4_^+^ from the developed method were compared with the standard analytical methods.

### 2.6. Analysis of NO_3_^−^ and NH_4_^+^ in Water and Soil Samples Using Portable Colorimetric Assays

The sample concentration of NO_3_^−^ and NH_4_^+^ was validated using portable colorimetric assays. For NH_4_^+^ detection, the commercial LCK 303 (HACH LANGE GMBH, Manchester, UK) was used as follows; the cap zip of the commercial tube was unscrewed and carefully removed the foil from the screwed-on cap zip. Then, 0.2 mL of sample was pipetted into the tube and the cap was immediately screwed back by fluting at the top. After that, the tube was shaken 2–3 times and left at room temperature for 15 min. Finally, the outside of the tube was cleaned with paper and placed into the reader. The method offered linearity in the range of 2.5–60.0 mg/L. For NO_3_^−^ detection, Palintest photometer 7100 (PHOT.23. AUTO, Gateshead, UK) was used. Briefly, the Nitratest tube was filled until 20.0 mL mark. One leveled spoon of Nitratest powder, and one Nitratest tablet was added; the tube was shaken for one minute and left for five minutes or longer to ensure complete settlement of powders and to obtain clear solution. The latter was carefully decanted into a round test tube and filled to 10.0 mL mark of tube. One Nitricol tablet was crushed and dissolved in 10.0 mL of clear solution. The tube was left for 10.0 min for color to fully develop. Finally, the tube was placed into the detector. The method allowed linearity over a range 0–20 mg/L of NO_3_^−^.

### 2.7. Selectivity Measurements

The selectivity was determined using separate solution method as suggested by Bakker [35]. Upon conditioning in 0.1 M of NaCl solution, electrode response was determined for each interfering ions separately. In the case of NO_3_^−^ response was collected in the following sequence of ions: Cl^−^, SO_4_^2−^, NO_2_^−^, OH^−^, NO_3_^−^, while in the case of NH_4_^+^, the sequence was: Na^+^, Ca^2+^, H^+^, K^+^, and NH_4_^+^.

## 3. Results and Discussion

### 3.1. The Role of ILs in ISE

**Figure 2:**
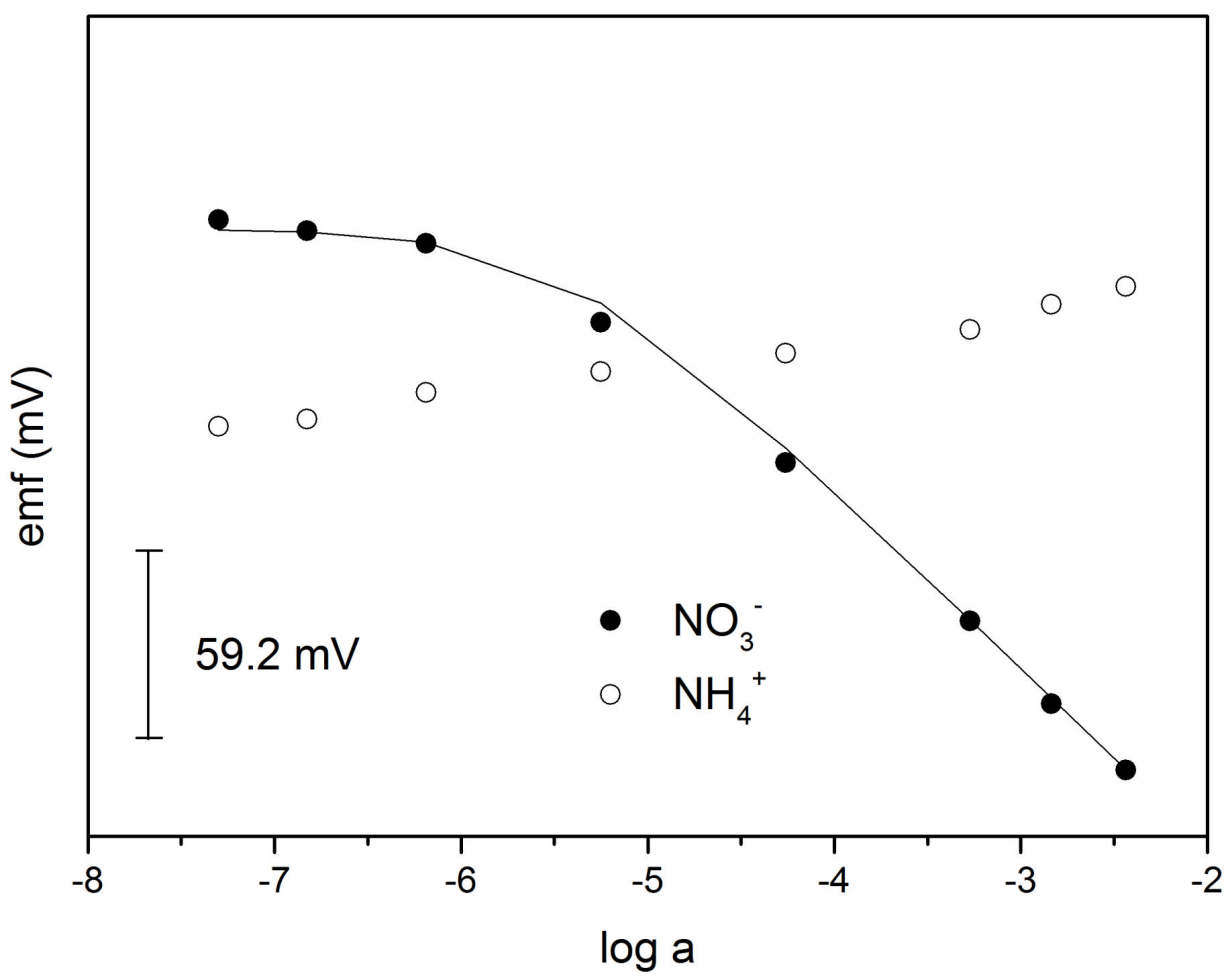
Response curves of NO_3_^−^- and NH_4_^+^-selective electrodes made of membranes 2 and 8 respectively.

In one of our previous works, we studied the influence of nine different ILs on selectivity on anion-selective electrodes [36]. In that work, we utilized ILs as plasticizers in ionopphore-free membranes and speculated that polarity of ILs was the key factor influencing selectivity. Building on that work, we decided to use IL-based membranes for development of NH_4_^+^- and NO_3_^−^-selective electrodes (membranes 2, 3, 8, and 9 from Table 1). In preparing NO_3_^−^-selective electrodes, we continued our practice of preparing ionophore-free membranes, which is also on accordance with common practice for preparing NO_3_^−^-selective electrodes. As expected, these electrodes showed Nernstian response slope and exhibits near-Nernstian slope with a detection limit of 5.5 × 10^−6^ M (0.2 ppm). The response of one such electrode can be seen in Figure 2 (full circles). However, the situation with NH_4_^+^-selective electrodes was not so straightforward. Namely, no electrode containing substantial amounts of ILs as plasticizer (membranes 8, 9, 10, and 11) showed Nernstian response. A typical response for NH_4_^+^-selective electrodes is seen in Figure 1 (open circles). It should be noted that the response was not well-defined, and the electrodes exhibited a substantial amount of drift. We then re-evaluated the response of electrodes prepared using classical compositions (membranes 1 and 7), and obtained expected responses (data not shown). These results prompted us to reconsider and rethink the role of IL in an ISE. It is known that ILs can partition into water as observed and carefully evaluated in the group of Kakiuchi [37]. We speculated that cations and anions from the ILs utilized in this study do not partition in water in equal measure due to significant differences in their lipophilicities. In other words, if the cation in the IL is significantly more lipophilic than the anion, the sensor will respond to NO_3_^−^, but not to NH_4_^+^, thus effectively behaving as ion exchange membrane. Indeed, when we prepared membranes containing 5 mmol/kg of IL and PVC-DOS—based matrix (membranes 5, 6), the response was anionic (data not shown). Therefore, this led us to conclude that although ILs can dissolve PVC and effectively behave as plasticizers, they have strong influence of functionality due to exchange of ions. Therefore, they are suitable as ion exchangers, but their use as plasticizers is severely limited and dependent on the specific IL.

### 3.2. MMA-DMA—Based ISE and Determination of NO_3_^−^ and NH_4_^+^ in Water and Soil Samples

In light of above discussion, it is clear that the ILs used in this study present significant limitations for describing cation-selective electrodes. To address this limitation, we decided to utilize acrylate-based membranes in preparation of NO_3_^−^ and NH_4_^+^ ISEs suitable for analysis of environmental samples. Membranes based on MMA-DMA have been utilized in the past due to excellent mechanical properties, reduced leaching of membrane components, and improved limit of detection [28,29]. We prepared membranes 12 and 13 as NO_3_^−^ and NH_4_^+^ ISEs respectively. The role of lipophilic salt (ETH 500) is required in order to reduce the resistance of membrane [20]. Electrodes exhibited near-Nernstian slopes and detection limits of 5.0 × 10^-6^ M (0.2 ppm) and 4.0 × 10^−6^ M (0.07 ppm) for NO_3_^−^ and NH^+^ electrodes respectively which corresponded nicely to classical PVC-DOS—based electrodes [17].

**Figure 3:**
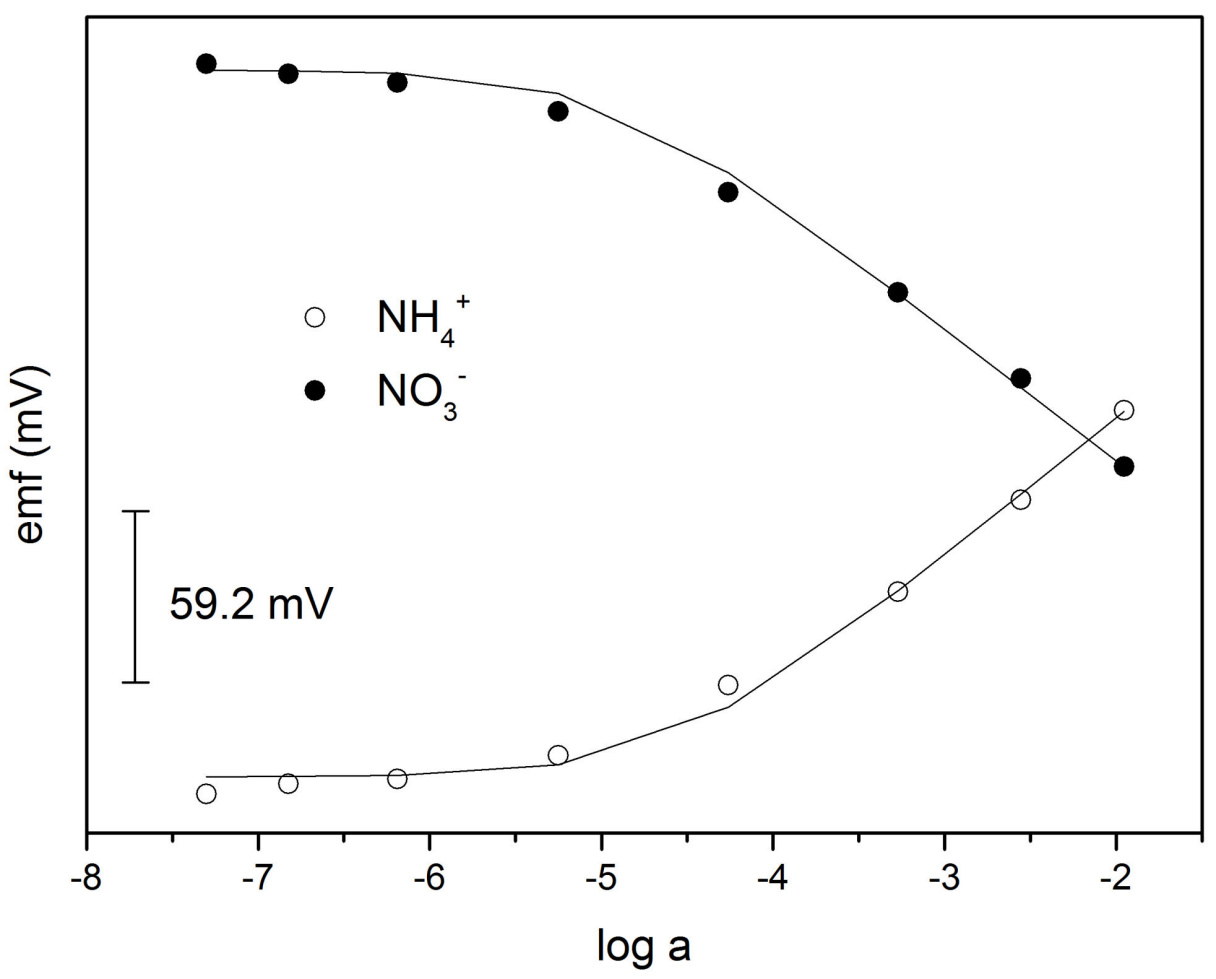
Response curves of NO_3_^−^- and NH_4_^+^ - selective electrodes. All electrodes are prepared on acetate sheet/graphite substrate. Linear fit of calibration points is obtained by fitting classical Nikolsky equation and experimentally available parameters for slope, selectivity coefficients, and sample composition.

Furthermore, Figure 3 shows potentiometric response of NH_4_^+^- and NO_3_^−^-selective electrodes to that are expected to be the most significant interferences in soil samples. Observation of near-Nernstian response slopes for all ions allowed us to calculate unbiased selectivity coefficients as shown in Table 3.

### 3.3. Application of MMA-DMA—Based ISEs in Environmental Analysis and Comparison to Colorimetric Assay

**Figure 4:**
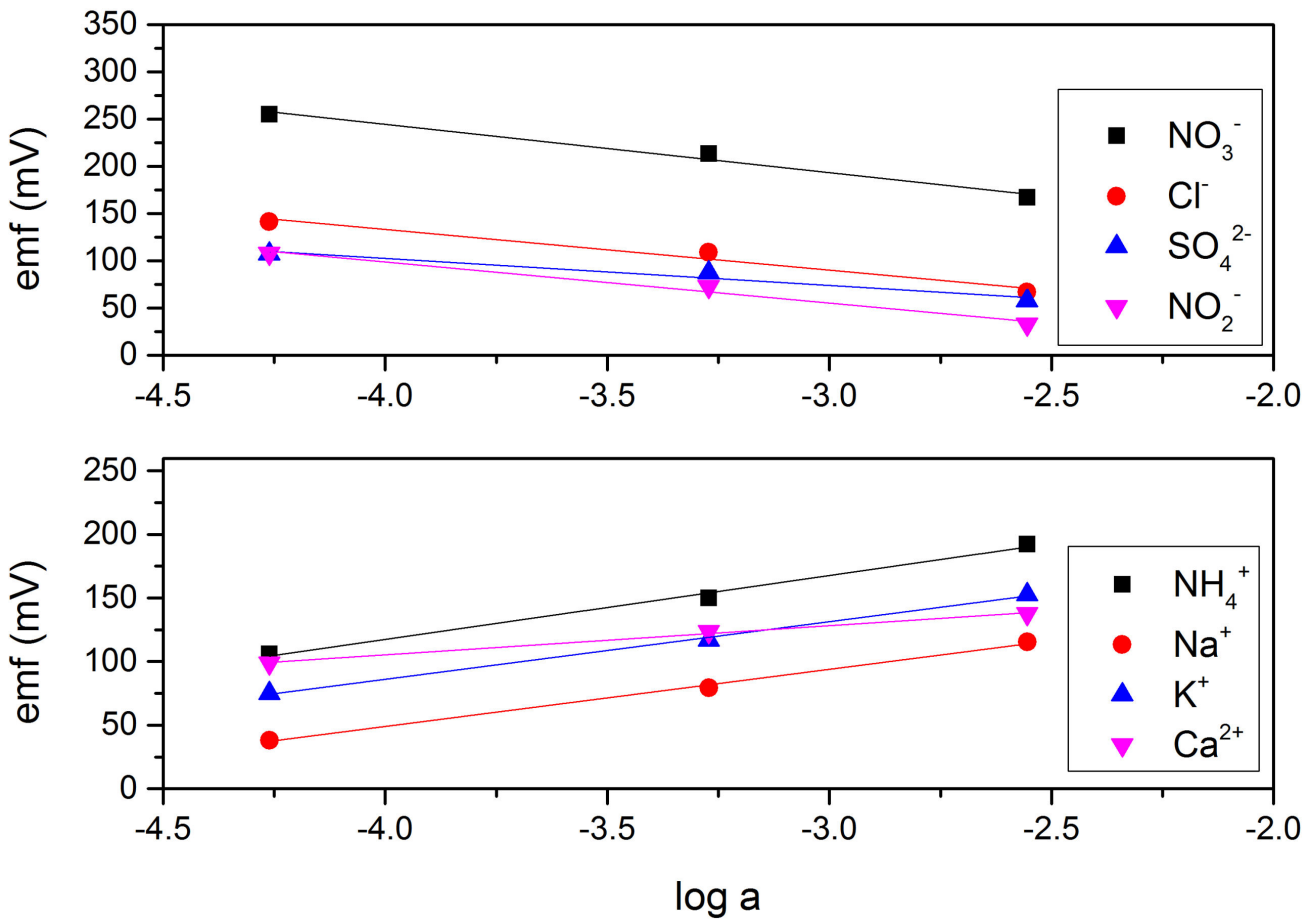
Potentiometric response for NO_3_^−^ (top)—and NH_4_^+^ (bottom)—selective electrodes to interfering ions.

**Figure 5:**
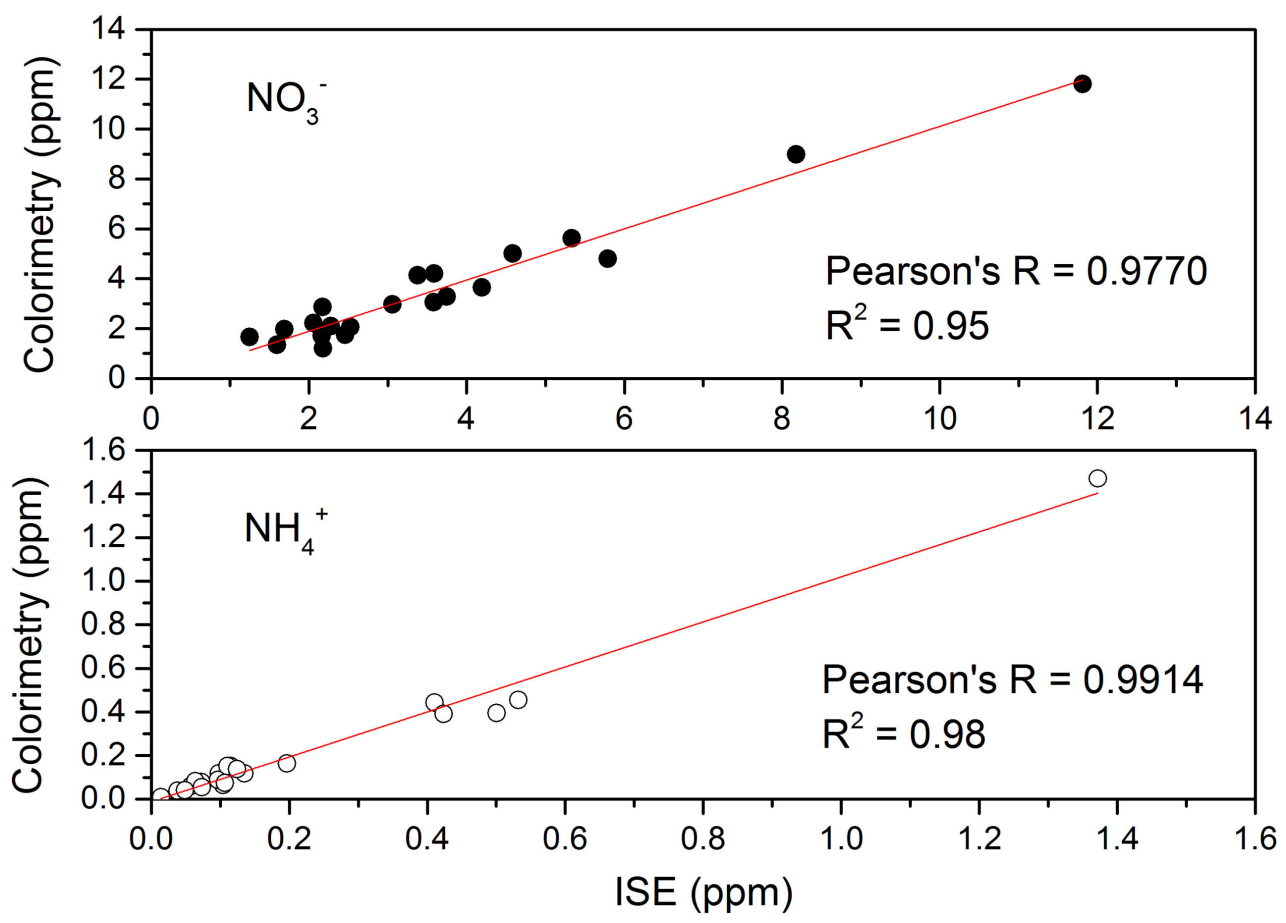
The comparison of the results obtained from determination of NO_3_^−^ (top) and NH_4_^+^ (bottom) in water and soil samples using the ISEs and portable techniques for 21 samples. In the case of ISEs, average values obtained from at least 3 different electrodes were used in this comparison.

Encouraged by these results, we decided to utilize MMA-DMA–based electrodes and determine concentration of NO_3_^−^ and NH_4_^+^ in real-life samples. We evaluated the results and procedures against colorimetric assays. The results are shown in Figure 4 and Figure 5.

We can see an excellent correspondence between the two techniques, indicating that ISEs can serve as alternative technique for simple determination of soil plant nutrients. However, let us compare and contrast procedures required for analysis by both techniques from the perspective of a non-trained person (e.g., farmer) and the convenience of the use on the field.

#### 3.3.1. Sample Preparation

For analysis of soil samples, analytes (in this case NH_4_^+^ and NO_3_^−^) have to first be extracted from the soil sample, typically by shaking of the sample in the presence of specified extractant (typically 1 M KCl).

*ISEs*: From ISEs perspective, highly concentrated extractant such 1M solutions are not suitable due to the currently insufficient selectivity of NH_4_^+^- and NO_3_^−^ -selective membranes. Thus, depending on the analyte and soil composition, a suitable extractant needs to be identified. Once a suitable extractant is identified, sample preparation for analysis by ISEs can be done in field.

*Colorimetric assays*: extraction of analyte must be followed by two-step filtering; initially through typical qualitative lab filter for removing bigger particles (e.g., Whatman Grade 1), followed by 0.45 microns syringe filters. This can be lengthy and tedious process, which is not realistically suitable for in field analysis.

#### 3.3.2. Sample Analysis

*ISEs*: Drawing analogy from pH measurements, sample analysis is done by a simple dipping of the electrode into the sample solution and collecting the reading.

*Colorimetric assays*: It can be seen from Section 2.6. that analysis of NH_4_^+^ involves a waiting step of 15 min, while analysis of NO_3_^−^ is even more complex, as it involves two steps: mixing the required chemicals with the sample, and waiting for pre-specified time. While this may not be problematic in the analysis of one sample, the length of analysis of multiple samples increases significantly. For example, in this work a fully trained PhD student took ~3.5 h to complete analysis of 46 samples (23 sample for each of NO_3_^−^ and NH_4_^+^ ions) in a fully equipped lab. Therefore, it is easy to imagine that field analysis of large area can occupy significant amount of time.

#### 3.3.3. Precision and Efficiency

*ISEs*: Dynamic range of ISEs can be multiple orders of magnitude, thus enabling the capture of wide concentration ranges. However, due to logarithmic dependence of signal versus the concentration, the precision is affected with pronounced negative effects if the concentration of the sample falls in the curvilinear response range [10]. The precision can be significantly improved if multiple ISEs of the same kind are used [38]. At present, only single ISEs are commercially available. However, due to the drive towards miniaturization, robustness, and simplicity of ISEs, it is easy to envision emergence of instrumentation containing multi-ISEs. This could be very important in the development of multi ion ISE assays, thus significantly simplifying while simultaneously improving precision of analysis.

*Colorimetric assays*: If the concentration in the sample falls in linearity range, the accuracy and precision of analysis is sufficient for routine analysis of nutrient level. However, if the concentration of the sample is outside of the range, the analysis complexity rises, as re-analysis is necessary. Furthermore, colorimetric assays are, by default, a single analyte analytical technique. Samples are analysed in a sequence of analytes.

## 4. Conclusions

Here, we have demonstrated that ILs with significant differences in polarity of cationic and anionic part can not be used as plasticizer. The more polar ion can be exchanged with the analyte, thus creating an ion exchanger. However, acrylate-based polymers are shown to be excellent materials for preparing plasticizer-free ISEs. We have prepared MMA-DMA—based, NH_4_^+^- and NO_3_^−^-selective ISEs using electrode substrates prepared using house-hold materials. We have demonstrated their functionality by analyzing 8 water and 15 soil samples. A comparison of results using ISEs and colorimetric assay showed excellent correlation (Pearson’s R = 0.97 and 0.99 for NO_3_^−^ and NH_4_^+^ ions respectively). Here, we have shown that due to the simplicity of operation and handling protocols, ISEs are in strong position to become the leading technique for quick and easy in field analyses, even by non-trained personnel.

## Figures and Tables

**Table 1:** Composition of membrane cocktails used in this work. Note that IL stands for both [P_6,6,6,14_][TFMS] and [P_6,6,6,14_][DCA]. In other words, two membranes, each with separate IL, are prepared.

Membrane	Ammonium I Ionophore	Additive (ETH 500)	Ion Exchanger		Plasticizer		Polymer
	Concentration (mmol/kg)	Concentration (mmol/kg)	Type	Concentration (mmol/kg)	Type	Concentration (wt%)	
1			TDACl	5	DOS	66	PVC
2,3			TDACl	5	IL	66	PVC
4,5			TDACl	5	DOS+IL	33 + 33	PVC
5,6			IL	5	DOS	66	PVC
7	10		NaTFPB	5	DOS	66	PVC
8,9	10		NaTFPB	5	IL	66	PVC
10,11	10		NaTFPB	5	DOS+IL	33 + 33	PVC
12		10	TDACl	5	N/A		MMA-DMA
13	10	10	NaTFPB	5	N/A		MMA-DMA

**Table 2:** Background soil analysis for soils collected at BIFoR. Results obtained are average for at least four replicates sampled for each sample location.

Soil Sample	pH	Moisture Content
		(g/g)
Ash	6.14 ± 0.04	0.35 ± 0.04
Oak	5.86 ± 0.02	0.29 ± 0.05
Scottish pine	6.03 ± 0.02	0.14 ± 0.03

**Table 3:** Selectivity coefficients for selected ions obtained for NH_4_^+^- and NO_3_^−^-selective electrodes using acetate sheet/graphite—based substrate and MMA-DMA based membrane composition.

NH_4_^+^-Selective Electrode			NO_3_^−^-Selective Electrode		
*Ion*	*Slope* ± *S.D.*	$logK_{I,J}^{pot}$ ± *S.D.*	*Ion*	*Slope* ± *S.D.*	$logK_{I,J}^{pot}$ ± *S.D.*
Na^+^	45.67 ± 1.0	−1.56 ± 0.01	Cl^−^	−42.89 ± 0.9	−2.24 ± 0.05
K^+^	46.88 ± 1.5	−0.65 ± 0.20	SO_4_^2−^	−24.82 ± 1.9	−2.59 ± 0.80
Ca^2+^	25.45 ± 2.6	−2.77 ± 0.20	NO_2_^−^	−42.80 ± 0.8	−2.83 ± 0.08
NH_4_^+^	52.96 ± 0.5	0	NO_3_^−^	−49.45 ± 1.3	0
